# Prognostic factors for continence after surgical correction of ectopic ureters of 51 dogs with long-term follow-up

**DOI:** 10.1186/s13028-022-00648-9

**Published:** 2022-11-17

**Authors:** Judith Visser, Hille Fieten, Hannah Wikje van Velzen, Marjanne Duke Zaal, Anne Kummeling

**Affiliations:** 1 Department of Clinical Sciences, Faculty of Veterinary Medicine, Utrecht University, Utrecht, The Netherlands; 2 Department of Dentistry, Oral and Maxillofacial Surgery, Eastcott Veterinary Referrals, Swindon, United Kingdom; 3 Veterinary Specialist Centre ‘De Wagenrenk’, Wageningen, The Netherlands

**Keywords:** Canine, Incontinence, Outcome, Prognosis, Surgery, Urology

## Abstract

**Background:**

An ectopic ureter is a congenital malformation characterized by caudal displacement of one or both ureteral orifices and is the most common cause of urinary incontinence in young dogs. Complete resolution of incontinence after surgery has been reported in 25–82% of dogs. The aim of this study was to identify preoperative prognostic factors for continence after surgical treatment of dogs with an ectopic ureter. Dogs were included if surgical correction of an ectopic ureter was performed and at least 1 year follow-up was available.

**Results:**

Fifty-one dogs met the inclusion criteria. The ectopic ureters were either intramural (91%) or extramural (9%). The ectopic ureters were bilateral in 49% of cases. Overall median follow-up time after surgery was 6.5 years (range 1–13 years). Surgical correction alone resolved urinary incontinence in 47% of cases. Low grade pre-operative incontinence, male sex and pre-operative presence of ureteral or renal pyelum dilation were significantly associated with urinary continence after surgery.

**Conclusions:**

Dogs with severe preoperative incontinence were less likely to become completely continent after surgery, whereas male sex and preoperative dilation of the ureter or renal pyelum were positive prognostic indicators for continence. These results may assist in predicting outcome after surgical correction of ectopic ureters and suggest assessment of pre-operative urethral pressure profiling in future studies.

## Background

Ureteral ectopia is a congenital abnormality of the urinary tract characterised by a caudal displacement of one or both ureteral orifices. The presence of an ectopic ureter (EU) is the most common cause for urinary incontinence in young dogs [1–3]. Ectopic ureters can be uni- or bilateral and are classified as intramural or extramural. Intramural EU, which are the most common type found in dogs [4], attach to the bladder near the trigone and then run submucosally in the bladder wall before terminating at a more caudal location. Extramural EU bypass the bladder entirely, attaching and emptying at any point caudal to the normal position and into the urethra [5, 6]. Ectopic ureter termination has been found in many different locations, including the bladder neck, prostatic urethra, pelvic urethra, vagina, and vestibule [5–7]. The severity of urinary incontinence, the most common clinical presentation of this abnormality, appears to be unrelated to EU characteristics (uni- or bilateral, intra- or extramural) [5].

Although surgical correction of EU is frequently performed, the outcome of surgery varies greatly, with reported rates of complete resolution of urinary incontinence varying between 25 and 82% [1, 2, 8–16]. Previous studies have investigated possible predictive prognostic factors for continence after surgery. In these studies, no significant association was identified between postoperative continence and sex [2, 17], EU characteristics (intra- vs extramural types) [1], age at surgery [9], preoperative urinary tract infection [9], pelvic bladder and other urogenital anatomic abnormalities [10, 14], neuter status [17], concurrent colposuspension [17] and surgical technique [1, 10, 11]. A recent study showed that cystoscopic-guided laser ablation had a significantly lower rate of recurrence of urinary incontinence when compared with neoureterostomy [16]. A study by Wiegand et al*.* [18] showed a better outcome in Labrador retrievers when compared to Golden retrievers, as well as a higher prevalence of preoperative hydronephrosis in Labrador retrievers. It remains challenging to predict long-term postoperative urinary continence for individual dogs, due to lack of standardisation of diagnostic and therapeutic protocols involved. The aim of this study was to evaluate potential prognostic factors associated with postoperative continence in a larger cohort of dogs with surgically corrected EU during a longer follow-up period (> 12 months).

## Methods

### Study group

Medical records from the teaching hospital, Utrecht University, The Netherlands, and the specialist referral centre, De Wagenrenk, Wageningen, The Netherlands, were screened retrospectively to identify dogs diagnosed with EU that were treated surgically between 2000 and 2019. Data retrieved from the medical records included breed, sex, age of onset of passive urinary incontinence, neuter status at time of surgery, age and bodyweight at time of surgery, characteristics of the EU (intra- or extramural, uni- or bilateral) and surgical technique. The presence of preoperative ultrasonographic ureteral or renal pyelum dilation was extracted from the imaging reports retrospectively. Upper urinary tract dilatation was documented if any fluid consistently remained visible in the ureter lumen or if the renal pelvis was dilated > 2 mm. Ultrasonographic imaging and interpretation was performed by ECVDI diplomates or ECVDI residents under direct supervision of an ECVDI diplomate.

### Follow-up

Owners of dogs with surgically corrected EU were interviewed by phone or e-mail. To determine outcome of surgery, owners were asked to score the severity of incontinence before and at a minimum period of 1 year after surgery on a six-point scale of 0–5, with 0 meaning no signs of urinary incontinence and 5 constant urinary leakage (Table 1). Outcome was determined to be successful when postoperative incontinence was absent, with or without additional medication (score 0). All other scores were considered to be non-effective.

**Table 1:** Scale for the scoring of incontinence in dogs with an ectopic ureter

Description	Score
No passive urinary incontinence	0
A few drops when the bladder is full	1
A few drops when the bladder is empty	2
A puddle when the bladder is full	3
A puddle when the bladder is empty	4
Permanent dribbling of urine	5

### Statistical methods

Statistical analysis was performed in ‘R’ (version 4.1.1, R Foundation for Statistical Computing, Vienna, Austria). Difference in presence of a dilated ureter or renal pyelum in Labrador retrievers versus other breeds and difference in age of onset of clinical signs of incontinence between males and females were tested with a Wilcoxon rank sum test with continuity correction. A P < 0.05 was considered significant.

Logistic regression was performed with outcome of surgery (post-operative urinary continence after a minimum postoperative period of 1 year versus any degree of postoperative urinary incontinence) as the dependent variable and breed (Labrador retriever versus other breeds), sex (male versus female), pre-operative dilation of the renal pyelum or ureter (absent versus present), neutering status at the time of surgery (intact versus neutered), pre-operative incontinence severity (scale 0–5), and characteristics of EU (unilateral versus bilateral) as independent variables. A univariable analysis was performed followed by determining the model of best fit determined by stepwise backward regression analysis, using Akaike’s information criterium.

## Results

### Dogs

Of the 103 dogs with follow-up  > 1 year that were identified from the medical records, 51 owners participated in the study (response rate 50%). All dogs presented for passive urinary incontinence (median score 5, range 2–5). The onset of urinary incontinence was noticed by owners at a median age of 9 weeks (range, 1 week–4 years). Onset of urinary incontinence was significantly earlier in females (median 60 days, range 7–518 days) when compared to males (median 140 days, range 28–1512 days) with P = 0.001.

Within the complete study group, 30 dogs were female (59%) and 21 dogs were male (41%). Of the male dogs, 19 were intact and 2 dogs were neutered simultaneously with EU surgery. Another 5 male dogs were neutered after surgical correction of EU. There were 25 intact female dogs and 5 spayed female dogs at the time of EU correction. Another 13 female dogs were spayed after EU correction. Of the 7 dogs that were neutered before surgery, 4 dogs showed urinary incontinence before neutering, 3 dogs developed urinary incontinence after neutering. Pre-operative incontinence scores in all 7 dogs neutered before surgery were based on severity of incontinence after neutering. Breeds represented included Labrador retriever (n = 24), Golden retriever (n = 5), Briard (n = 3), mixed-breed dogs (n = 4), English bulldog (n = 2) and one each of the following breeds: American bulldog, American Staffordshire terrier, Australian terrier, Beagle, Border collie, Dutch kooiker dog, Dutch partridge dog, Entlebucher mountain dog, Jack Russell terrier, Landseer, Pit Bull, Welsh springer spaniel and Welsh terrier.

### Diagnostic findings

An ectopic ureter was present unilaterally in 26 dogs (51%) and bilaterally in 25 dogs (49%). One of the bilaterally affected dogs presented with both an intramural and an extramural EU. Overall, 91% of EU were intramural, while 9% were extramural. Ectopic ureters were diagnosed by abdominal ultrasonography (n = 36), computed tomographic excretory urography (n = 4), radiographic excretory urography (n = 3), retrograde urethrocystoscopy (n = 2) or exploratory surgery (n = 1). Mode of diagnosis was not recorded for the remaining 5 cases. Preoperative dilation of the ureter or renal pyelum was not recorded in 2 cases, in the remaining 49 dogs, dilatation was present in 55% (27 cases) and was not significantly different between the Labrador retriever and other breeds.

### Treatment and outcome

Surgical correction of EU was performed between May 2000 and June 2019. All surgeries were performed by ECVS diplomates, ECVS residents under direct supervision of an ECVS diplomate, or ECVS residency-trained surgeons. A total of 76 EUs were surgically corrected; 63 intramural EUs were treated by neoureterostomy, the other intramural cases were corrected by cystoscopic-guided laser ablation (1 ureter), ureteral reimplantation (1 ureter) or ureteronephrectomy (2 ureters). Mode of correction was not recorded for 2 intramural ureters. All extramural EUs (n = 7) were corrected by ureteral reimplantation. Median bodyweight at the time of surgery, recorded for 43 dogs, was 22 kg (range 4–62 kg) and median age at surgery was 10 months (range 51 days–6.7 years). The median age at time of surgery was significantly lower in female dogs (median 15 months, range 51 days–6.7 years) than in male dogs (28 months, range 4 months–6.3 years) with P = 0.008. Long-term follow-up (> 1 year) was not available for 2 dogs, due to euthanasia performed at 36 and 133 days after surgery respectively after surgery because of persistent severe urinary incontinence; their data were included in the analysis. The median follow-up period of the remaining 49 dogs was 6.5 years (range, 1–13 years). Twenty-four of the 51 dogs were continent after surgery alone with a minimum postoperative period of 1 year, resulting in a successful long-term outcome in 47% of dogs. Another 3 dogs were continent with the addition of medication to control passive urinary incontinence (2 dogs phenylpropanolamine only, 1 dog both phenylpropanolamine and estriol), resulting in an overall success rate of 53%. Results of univariable analysis and model of best fit are included in Table 2.

**Table 2:** Results of the logistic regression analyses of potential prognostic variables

Independent variable	Univariable analysis		Model of best fit (AIC = 63.63)	
	OR (95% CI)	P	OR (95% CI)	P
Sex (male (ref) vs female)	3.75 (1.18–13.17)	0.03*	5.76 (1.41–31.04)	0.023*
Breed (Labrador retriever (ref) vs other breeds)	0.8 (0.26–2.42)	0.70		
Preoperative incontinence (scale 1–5)	1.69 (1.02–3.05)	0.055	1.83 (1.013–3.69)	0.061
Location (bilateral (ref) vs unilateral	0.68 (0.22–2.04)	0.49		
Preoperative ureteral/pyelum dilation (absent (ref), present)	0.49 (0.15–1.52)	0.22	0.18 (0.031–0.74)	0.029*
Castration status (intact (ref) vs neutered)	3.29 (0.63–24.75)	0.18		

The dependent variable surgical outcome was defined as 0 = continent  > 1 year after surgery, 1 = incontinent  > 1 year after surgery

Reference category is indicated with (ref)

^*^significant; *OR* Odds ratio, *CI* confidence interval, *AIC* Akaike’s information criterium

## Discussion

Surgical correction of EU alone had a successful outcome, defined as complete long-term urinary continence, in 47% of cases in this study population. This is in accordance with earlier studies reporting postoperative incontinence rates varying between 25 and 82% [1, 2, 8–11, 13–15]. Three dogs still required medication after surgery to remain continent, with an overall success rate of 53% for long-term continence.

In the model of best fit, ureteral or renal pyelum dilation and male sex were significantly associated with a higher chance of becoming continent post-operatively, whereas a more severe pre-operative incontinence score was a negative prognostic indicator for postoperative continence.

It has been reported that Siberian huskies have a significantly lower and Labrador retrievers a significantly higher rate of successful outcome of surgery [2, 9, 18]. Labrador retrievers did not have a better outcome than any of the other breeds included in our study. No Siberian huskies were included in our study population. An interesting finding in the study of Weigand et al*.* [18] was that Labrador retrievers more often presented with a dilated ureter or renal pyelum than other breeds. This was not confirmed in our study. It has been suggested that normal vesico-urethral sphincter function could cause more pronounced ureteral or pyelum dilation in dogs with EU. An ectopic ureter passing through a well-functioning vesico-urethral sphincter may be prone to become temporarily or partially obstructed leading to hydroureter or dilated renal pelvis, as was hypothesised by Wiegand et al*.* [18]. Good sphincter function is probably beneficial after correction of the EU, where it contributes to the continence of the dog post-surgically. The results of our study fit this hypothesis, where dogs with pre-operative dilation of ureters or renal pelvis had a higher chance of becoming continent post-operatively.

Preoperative urethral pressure profiling was previously reported to be a potential useful tool in predicting postoperative incontinence due to concurrent functional abnormalities of the urinary tract, such as urinary sphincter mechanism incompetence [19, 20]. However, this has not been validated in a larger group of dogs. In future studies, it would be interesting to assess urethral pressure profiling values in combination with incontinence severity before and after surgery.

The results of the study from Wiegand et al*.* [18] also reported that female dogs have a higher risk of postoperative incontinence than males, which was confirmed in our study, although another study [2] did not find a difference. Due to increased urethral length in male dogs, passive urinary incontinence may not be apparent or only present later in life. Therefore, the prevalence of ureteral ectopia in male dogs may be underestimated and is often diagnosed at an older age. Male dogs may also have a better clinical outcome after surgery due to this increased length [2, 10, 21]. The realisation that especially male dogs with EU may remain asymptomatic or do not show urinary incontinence until a later age, has led to an increase in the number of male dogs being diagnosed with EU [2, 22]. This is reflected in a different female to male ratio found in this study, which was approximately 2.1:1, compared to the ratio reported by Wiegand et al*.* [18], who showed a female to male ratio of 6.2:1. This higher prevalence is likely due to increased education as well as improved diagnostic modalities.

The severity of urinary incontinence, subjectively scored by the owner, was a negative prognostic factor for continence after surgery for EU in our study. This result seems logical, but recall bias may be a limitation of subjective scoring of incontinence by owners, due to the retrospective study design. It is possible that owners of dogs in which surgery was effective, scored preoperative severity of incontinence differently compared to owners of dogs that remained incontinent after surgery. The severity of urinary incontinence may also show variation due to fluctuations in water uptake, concurrent bacterial infections, medical treatment and oestrous cycle. Despite these limitations, when incontinence is observed over a longer period of time, the owners’ perception of the severity of incontinence becomes more reliable and clinically relevant. In future studies, a validated scoring system of urinary incontinence should be used prospectively in order to confirm the findings of this study.

Neutering before or after EU correction has been discussed as a risk factor for urinary incontinence in dogs with EU [23]. A recent publication by Hoey et al*.* [15] did not show a significant increase in incontinence scores if neutering of female dogs was performed after surgical correction of EU, but in the study of Dekerle et al. [16], 3 of the 5 dogs that were spayed after EU correction had recurrence of urinary incontinence. Results of our study did not show a significant increase in the risk of postoperative urinary incontinence in dogs with EU that were already neutered at the time of surgical correction. Interestingly, 3 intact dogs started showing signs of urinary incontinence related to EU only after they were neutered. Neutering may have been an additional causative factor in the development of urinary incontinence in these dogs, as it can decrease urethral tone [23]. However, the number of dogs that were neutered either before or after EU correction may have been too small to identify neuter status as a prognostic factor (type 2 error).

Although recently endoscopic laser ablation was identified as a technique with a more favourable outcome [16], surgical technique was not included as a dependent variable in this study because only 1 of 69 intramural EU was treated with laser ablation. Although the choice of surgical technique is often elective in intramural EU and not specifically case related, in the future prediction modelling surgical technique should be considered as a confounder for outcome.

## Conclusions

Female dogs and dogs with more severe preoperative incontinence were less likely to become completely continent after surgery, whereas preoperative dilation of the ureter or renal pyelum was associated with a higher chance of continence after surgery. Preoperative ureteral or renal pyelum dilation may result from vesico-urethral sphincter contractions on the ectopic part of the ureter. In future studies, assessment of urethral pressure profiling before surgery of EU may be of interest for predicting successful outcome of surgery.

## Data Availability

The datasets used and/or analysed during the current study are available from the corresponding author on reasonable request.
